# Development of a multiple-gene-loading method by combining multi-integration system-equipped mouse artificial chromosome vector and CRISPR-Cas9

**DOI:** 10.1371/journal.pone.0193642

**Published:** 2018-03-05

**Authors:** Kazuhisa Honma, Satoshi Abe, Takeshi Endo, Narumi Uno, Mitsuo Oshimura, Tetsuya Ohbayashi, Yasuhiro Kazuki

**Affiliations:** 1 Department of Biomedical Science, Institute of Regenerative Medicine and Biofunction, Graduate School of Medical Science, Tottori University, Yonago, Tottori, Japan; 2 Chromosome Engineering Research Center, Tottori University, Yonago, Tottori, Japan; 3 Tottori Industrial Promotion Organization, Tottori, Tottori, Japan; 4 Division of Laboratory Animal Science, Research Center for Bioscience and Technology, Tottori University, Yonago, Tottori, Japan; Cleveland Clinic, UNITED STATES

## Abstract

Mouse artificial chromosome (MAC) vectors have several advantages as gene delivery vectors, such as stable and independent maintenance in host cells without integration, transferability from donor cells to recipient cells via microcell-mediated chromosome transfer (MMCT), and the potential for loading a megabase-sized DNA fragment. Previously, a MAC containing a multi-integrase platform (MI-MAC) was developed to facilitate the transfer of multiple genes into desired cells. Although the MI system can theoretically hold five gene-loading vectors (GLVs), there are a limited number of drugs available for the selection of multiple-GLV integration. To overcome this issue, we attempted to knock out and reuse drug resistance genes (DRGs) using the CRISPR-Cas9 system. In this study, we developed new methods for multiple-GLV integration. As a proof of concept, we introduced five GLVs in the MI-MAC by these methods, in which each GLV contained a gene encoding a fluorescent or luminescent protein (EGFP, mCherry, BFP, Eluc, and Cluc). Genes of interest (GOI) on the MI-MAC were expressed stably and functionally without silencing in the host cells. Furthermore, the MI-MAC carrying five GLVs was transferred to other cells by MMCT, and the resultant recipient cells exhibited all five fluorescence/luminescence signals. Thus, the MI-MAC was successfully used as a multiple-GLV integration vector using the CRISPR-Cas9 system. The MI-MAC employing these methods may resolve bottlenecks in developing multiple-gene humanized models, multiple-gene monitoring models, disease models, reprogramming, and inducible gene expression systems.

## Introduction

There are several concerns about conventional gene delivery vectors, namely plasmids, bacterial artificial chromosomes (BACs), and P1-derived artificial chromosomes (PACs), for the production of stable transgenic (Tg) cells and animals, such as unpredictable copy number, disruption of the host genome by random integration, transgene silencing by position effect, and limitation of gene-loading size [1]. Therefore, alternative tools for resolving these problems are strongly desired. Previously, we developed a human artificial chromosome (HAC) vector from native human chromosomes by chromosome engineering, telomere-associated chromosomal truncation, and loxP site insertion [2, 3]. The HAC vector has different properties from those of other gene delivery vectors, for example delivery of a defined copy number of transgene, stable and independent maintenance in host cells without integration, transferability from donor cells to recipient cells via microcell-mediated chromosome transfer (MMCT), and the potential for loading a megabase (Mb)-sized DNA fragment [4]. Additionally, since the HACs have a loxP site for site-specific recombination (SSR), gene-loading vectors (GLVs) carrying a loxP site can be integrated efficiently.

Using the advantages of the HAC, we have established various transgenic cells for gene function analysis, differentiation monitoring systems, and gene and cell therapy [5, 6]. We have also developed various HACs holding a huge DNA fragment; examples of this include a HAC carrying the human CYP3A cluster (about 0.7 Mb) for humanized model mice and a HAC carrying 2.4 Mb of the whole dystrophin gene for gene therapy [4, 7, 8]. Although the HAC is retained in human-derived cells at high efficiency, the retention rate varies among mouse tissues; in particular, hematopoietic cells showed a low retention rate. Therefore, we have developed a mouse artificial chromosome (MAC) vector from a native mouse chromosome in the same way as used for HAC construction. In addition to the advantages of the HAC, the MAC has a high retention rate in mouse tissues including hematopoietic cells [9, 10]. The MAC is also stably maintained in human cells in vitro upon long-term culture [10]. Therefore, the MAC is an extremely useful vector similar to the HAC, which also overcomes the disadvantages of other GLVs. However, the HAC/MAC only has a loxP site for gene loading, so the labor-intensive process of additional GLV loading must be performed.

Multiple-GLV-loading systems are expected to promote multiple-gene humanized models, multiple-gene monitoring models, disease models, reprogramming, and inducible gene expression systems. To expand the range of applications of the HAC/MAC, we have developed the Sequential or Simultaneous Integration of a Multiple-GLV (designed as the SIM)-loading system, involving two different approaches: the sequential integration method and the simultaneous integration method. Both approaches have common advantages, such as high efficiency of the gene targeting by SSR systems (Cre-loxP, φC31 and Bxb1 integration system), the theoretically unlimited number of GLVs that can be loaded by reusing two drug resistance genes (DRGs) and two SSR systems, and the applicability to the HAC/MAC with a loxP site [11]. However, the SIM system employs the HPRT gene reconstitution system to clone the GLV into the HAC/MAC via HAT selection. Then, in this system, GLV loading needs to be carried out in HPRT-deficient cells.

Previously, we developed a multi-integrase (MI) system for loading multiple GLVs on the HAC/MAC (MI-HAC/MI-MAC) [9, 12]. The MI system uses five SSR sites: four irreversible integration systems (TP901, Bxb1, φC31, and R4 integration systems) and one reversible recombination system (FRT/Flp recombination system). Yoshimura et al. (2015) reported that the MI-MAC was transferred to mouse embryonic stem cells (mESCs) in which the HPRT gene is wild type as target cells via MMCT prior to loading a GLV. The efficiency of GLV integration into each SSR site on the MI-MAC was >50% in mESCs and it enables the convenient production of transchromosomic mice with a gene of interest (GOI). Therefore, the establishment of various cell lines carrying the MI-MAC reduces the time and labor required for GLV loading because these cells can be used as host cells without knocking out the HPRT gene and MMCT. Then, to load a GLV possessing a GOI onto MI-MACs in desired cells, only conventional transfection methods that have been adapted to the desired cells are needed. Furthermore, the MI-MAC can carry large genomic DNA, for example, two PACs (29 and 39 Kb), for monitoring toxicity to cells [13]. However, in the original MI system, the DRGs cannot be reused repeatedly. That is, the MI-MAC is theoretically capable of holding five GLVs, but the rest of usable DRGs for selection is reducing as GLV-loading with drug selection. Then the limitation of the DRGs for the selection restricts the function of the MI-MAC as a multiple-GLV-loading system.

Recently, genome editing techniques using the zinc finger nuclease (ZFN), the transcription activator-like effector nuclease (TALEN), and the clustered regularly interspaced short palindromic repeat and CRISPR-associated protein (CRISPR-Cas9) have rapidly advanced [14–16]. Their common function is the induction of a double-strand break (DSB), which is then repaired by either homology directed repair (HDR) or non-homologous end joining (NHEJ). NHEJ involves error-prone machinery, resulting in gene disruption by an insertion or deletion (indel) of DNA. Therefore, genome editing technology is efficient for the induction of gene disruption. Among these genome editing techniques, the CRISPR-Cas9 system may have received the most attention because of its simplicity of construction.

**Fig 1:**
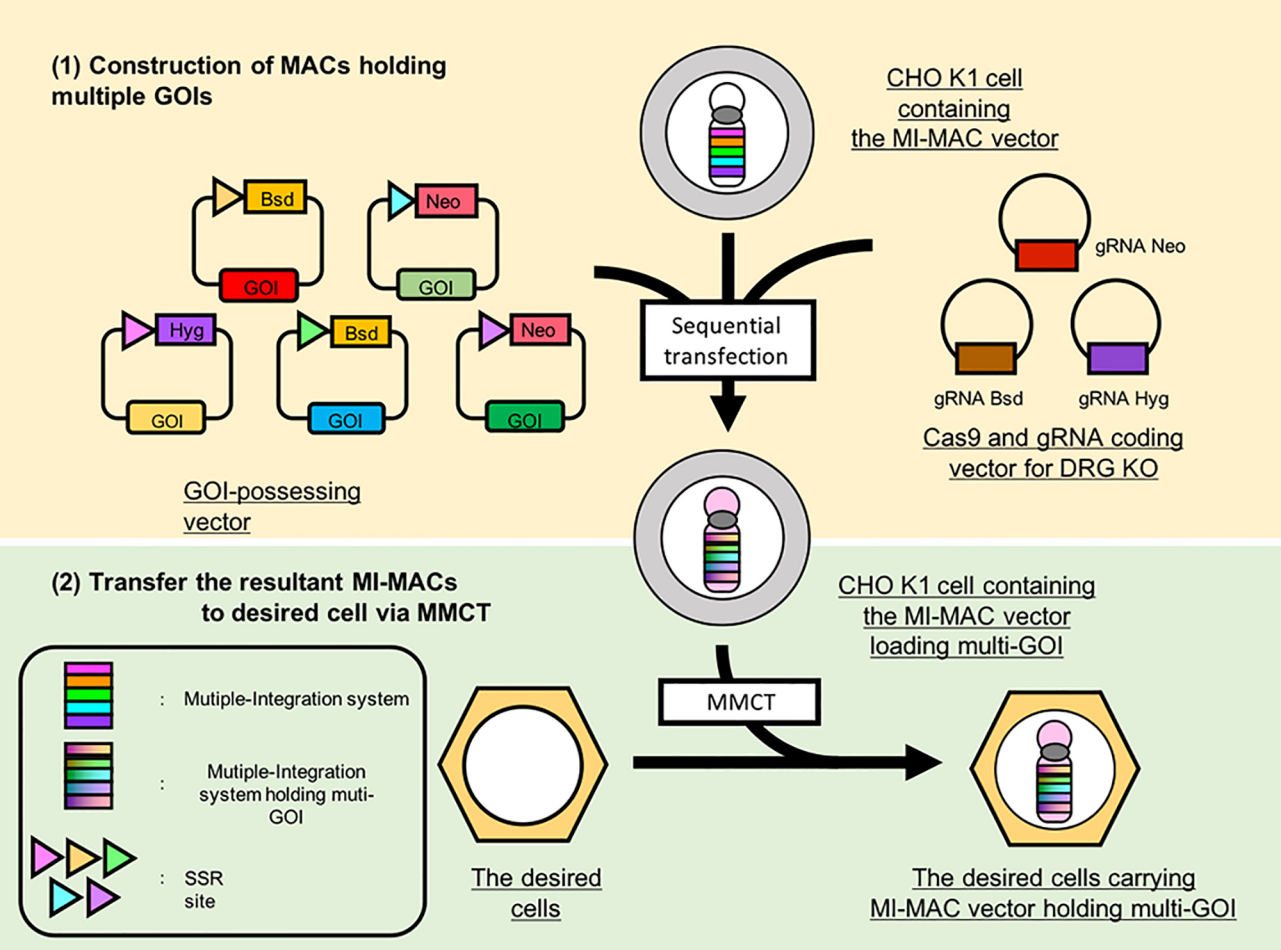
Schematic diagram of multiple-GLV loading on MI-MACs in Chinese Hamster Ovary (CHO) K1 cells and establishment of desired cells carrying the resultant MAC. (1) Construction of MI-MACs carrying multiple GOIs. Multiple GLVs are loaded on mouse artificial chromosome (MAC) vectors equipped with the MI system and the resultant MI-MAC is constructed. Each GOI-possessing vector is integrated using the integration/recombination systems of the MI-MAC. gRNAs target drug resistance genes (blasticidin resistance gene: Bsd, neomycin resistance gene: Neo, hygromycin resistance gene: Hyg) for gene KO to reuse these genes repeatedly. (2) The resultant MI-MACs carrying multiple GOIs are transferable to various cell lines. The resultant MI-MACs are transferred to the desired cells. via microcell-mediated chromosome transfer (MMCT).

In this study, we attempted to reuse DRGs using the CRISPR-Cas9 system and to integrate multiple GLVs onto the MI-MAC (Fig 1). We developed methods for multiple-GLV loading on the MI-MAC. As a proof of principle, we integrated five GLVs into five SSR sites on the MI-MAC in Chinese Hamster Ovary (CHO) K1 cells in which the HPRT gene is the wild type, and each of these GLVs contained a gene encoding a fluorescent or luminescent protein, EGFP, mCherry, BFP, enhanced green-emitting luciferase (Eluc) and Cypridina luciferase (Cluc) [17, 18]. The resultant MI-MACs were successfully transferred to HT1080 (human fibrosarcoma) cells via MMCT. The HT1080 cells carrying the MI-MAC exhibited the five fluorescence/luminescence signals.

## Materials and methods

### Cell culture

CHO K1 cells were cultured in Ham’s F-12 medium (Wako) supplemented with 10% fetal bovine serum (FBS, Corning), 100 units of penicillin–streptomycin (PS, Wako). CHO K1 cells carrying the MAC1 (CHO MAC1) were maintained with 800 μg/mL G418 (Promega). CHO cells carrying the MAC2 (CHO MAC2) were maintained with 500 μg/mL hygromycin (Hyg, Wako). CHO cells carrying the 21HAC2 (CHO 21HAC) were maintained with 8 μg/mL blasticidin S (BS, KNF). CHO K1 cells carrying the MI-MAC were maintained with 200 μg/mL hygromycin. CHO K1 cells carrying the MI-MAC(1) the MI-MAC(2), and the MI-MAC(3)+CrB were maintained with 600 μg/mL G418. CHO K1 cells carrying the MI-MAC(3), the MI-MAC(4), and the MI-MAC(2)+CrN were maintained with 6 μg/mL blasticidin S. The CHO K1 cells carrying the MI-MAC(5) or the MI-MAC(5) Sim were maintained with 500 μg/mL hygromycin. The HT1080 cells were cultured in Dulbecco’s modified Eagle’s medium (Wako) supplemented with 10% FBS and 100 units of PS. The HT1080 cells carrying the MI-MAC(5) or the MI-MAC(5) Sim were selected with 800 μg/mL G418. The cells were cultured in an incubator humidified at 37°C/5% CO_2_.

### Plasmid construction

For construction of the TP901neo CAG-EGFP vector for loading of the TP901-1 attP on the MI-MAC, a TP901 attB cassette of the TP901neo inspB4ins2 plasmid was digested with ScaI and NheI (1.1 kb) and inserted at the ScaI and NheI site (11.7 kb) of the φC31neo CAG-Eluc backbone vector [19]. For construction of the Bxb1neo CAG-EGFP vector, AcsI- and AvrII-digested synthetic oligonucleotides (1.3 kb) containing a Bxb1 attB-Neo cassette were inserted into the AscI and NheI site (10.7 kb) of the ins2 CAG-EGFP backbone plasmid [19]. For construction of the φC31bsd CAG-mCherry vector, a φC31 attB-Bsd cassette in the φC31bsd inspB4ins2 plasmid was digested with AscI and NruI (1.0 kb) and inserted into the ScaI and NheI (10.6kb) site of the ins2 CAG-mCherry backbone vector. For construction of the R4bsd CAG-BFP vector, a BFP cassette of the pTag-BFP plasmid was digested with BamHI and NotI (0.7 kb) and inserted into the BamHI and NotI site (10.7 kb) of the R4bsd CAG-EGFP backbone vector. The FRThyg CAG-Cluc vector was constructed as follows: a Hyg-HS4 cassette of the Bxb1hyg CAG-EGFP plasmid was digested with NheI and SnaBI (4.3 kb) and inserted into the NheI and SnaBI site (5.6 kb) of the FRTneo CAG-EGFP backbone vector, then a CAG-Cluc cassette of the φC31neo CAG-Cluc plasmid was digested with SnaBI and NotI (3.1 kb) and inserted into the SnaBI and NotI site (9.9 kb) of the FRTneo CAG-EGFP backbone vector [19].

The gRNA/Cas9 co-expression vectors were designed using the Optimized CRISPR Design site (http://crispr.mit.edu) and made by loading the synthetic oligonucleotides. Sense and antisense synthetic oligonucleotides of gRNA coding sequences were subcloned into the Bbs I site of the pX330 vector after annealing. Plasmid vector pX330-U6-Chimeric_BB-CBh-hSpCas9 was a gift from Feng Zhang (Addgene plasmid # 42230) [20].

### MMCT

The MI-MAC vectors were transferred from the mouse fibroblast(A9) cells to the CHO K1 cells using PEG-MMCT technology for the establishment of the recipient cells to load multiple GLVs [13, 21, 22]. The CHO K1 hybrids were selected in 500 μg/mL hygromycin. The MI–MAC(5) and MI-MAC(5) Sim vectors were transferred from the CHO K1 cells into the HT1080 cells using MV-MMCT technology [23]. The HT1080 hybrids were selected in 800 μg/mL G418.

### Transfection of the CHO K1 cells

For the evaluation of the frequency of mutation induction by each gRNA, all transfections were carried out in CHO cells carrying MAC1, MAC2 or 21HAC2 by lipofection (Lipofectamine LTX, Invitrogen), in accordance with the manufacturer’s instructions. The gRNA/Cas9 co-expression vector was transfected at the maximum level as described manufacture ‘s instructions. At 3days after transfection, genomic DNA was extracted and evaluated.

For the multiple gene loading experiments, all transfections in the method which gene loading and disruption of DRG were performed singly, were carried out in the CHO K1 cells carrying each MI-MAC. GLV loading onto each MI-MAC was carried out by lipofection, in accordance with the manufacturer’s instructions and as described above [12]. The details of the MAC vector’s name, the transgene’s name, and acquired resistance genes used in the method are shown in Table 1. For the transgene integration, the weight ratio of the transgene and the integrase-expression vector was 3–4:1. These cells were seeded on 10-cm dishes at 1 day post-transfection. Then, drug selection was started at 2 days post-transfection. At 10–11 days after transfection, clones were picked up. For the DRG knock-out, the gRNA/Cas9 co-expression vector was transfected at the maximum level as described in the manufacturer’s instructions. These cells were seeded on 10-cm dishes at 4–5 days post-transfection. At 14–15 days after transfection, clones were picked up and separated into two wells. These cells were exposed to two different selection drugs. In one group in this experiment, culturing was performed under the same conditions as prior to the clones being picked up for maintenance, while in the other, supplementation with the drug corresponding to each gRNA targeted for the detection of the knockout was performed. Specifically, the CHO K1 clones carrying the MI-MACΔ(1), the MI-MACΔ(3), and the MI-MACΔ(4) were maintained with appropriate drugs (200 μg/mL Hyg, 600 μg/mL G418, and 6 μg/mL BS, respectively) to detect GLV loading; alternatively, selection with these drugs (200 μg/mL Hyg + 600 μg/mL G418, 6 μg/mL BS + 600 μg/mL G418, and 500μg/mL Hyg, respectively) was performed to evaluate the knockout of the DRG. The drug-sensitive cells were analyzed in the following experiments.

**Table 1:** The characteristics of the MI-MACs, the transgenes, acquired resistance genes, knock-out frequency and integration frequency in the original method.

Transfection number	MAC vector name	Transgene name	Acquired resistance genes	Knockout frequency	Integration frequency
0	MI-MAC	—	—	—	—
1	MI-MAC(1)	TP901-1neo CAG-Eluc	Hyg	—	41.7% (10/24)
			Neo		
2	MI-MACΔ(1)	gRNA Neo	Hyg	3.1% (6/192)	—
3	MI-MAC(2)	Bxb1neo CAG-EGFP	Hyg	—	91.7% (22/24)
			Neo		
4	MI-MAC(3)	φC31bsd CAG-mCherry	Hyg	—	100% (24/24)
			Neo		
			Bsd		
5	MI-MACΔ(3)	gRNA Bsd	Hyg	16.7% (4/24)	—
			Neo		
6	MI-MAC(4)	R4bsd CAG-BFP	Hyg	—	4.2% (2/48)
			Neo		
			Bsd		
7	MI-MACΔ(4)	gRNA Hyg	Neo	7.3% (7/96)	—
			Bsd		
8	MI-MAC(5) Seq	FRThyg CAG-Cluc	Neo	—	29.2% (7/24)
			Bsd		
			Hyg		

The method for sequential integration with simultaneous disruption of DRG was carried out by lipofection as described above in CHO K1 cells [12]. The details of the MAC vector name, transgene name, and acquired resistance gene used in the method are shown in Table 2. For the transgene integration, the weight ratio of the transgene and the integrase expression vector was 4:1. For the transgene integration and the DRG knockout simultaneously, the weight ratio of the transgene, the integrase expression vector, and gRNA/Cas9 co-expression vector was 5:2:4. The cells were seeded on 10-cm dishes at 1–5 days post-transfection. After 8–15 days of selection, drug-resistant clones were picked up and cultured in two wells separately under different drug conditions: one involved culture to enrich and increase the cells expected to be loaded with GLVs. The other was performed to detect the cells with DRG knockout corresponding to each transfected gRNA. Specifically, the CHO K1 clones carrying the MI-MAC(2)+CrN, the MI-MAC(3)+CrB, and the MI-MAC(4)+CrH were selected with drugs (6 μg/mL BS, 600 μg/mL G418, and 6 μg/mL BS, respectively) to obtain the clones in which GLV loading had occurred as expected; alternatively, supplementation with these drugs (600 μg/mL G418 + 6 μg/mL BS, 600 μg/mL G418 + 6 μg/mL BS, and 500 μg/mL Hyg, respectively) was performed to evaluate knockout of the DRGs. PCR analyses were carried out to confirm the correct loading of the DRGs into the SSRs.

**Table 2:** The characteristics of the MI-MACs, the transgenes, acquired resistance genes, knock-out frequency and integration frequency in the improved method.

Transfection number	MAC vector name	Transgene name	gRNA name	Acquired resistance genes	Knockout frequency	Integration frequency
0	MI-MAC	—	—	Hyg	—	—
1	MI-MAC(1)	TP901neo CAG-Eluc	—	Hyg	—	41.7% (10/24)
				Neo		
2	MI-MAC(2) + CrN	φC31bsd CAG-mCherry	gRNA Neo	Hyg	7.8% (15/192)	100% (15/15)
				Bsd		
3	MI-MAC(3) + CrB	Bxb1neo CAG-EGFP	gRNA Bsd	Hyg	21.4% (6/28)	100% (6/6)
				Neo		
4	MI-MAC(4) + CrH	R4bsd CAG-BFP	gRNA Hyg	Neo	88.2% (15/17)	26.7% (4/15)
				Bsd		
5	MI-MAC(5) Sim	FRThyg CAG-Cluc	—	Neo	—	12.5% (3/24)
				Bsd		
				Hyg		

### Genomic PCR analysis

Genomic DNA was extracted from cell lines using Puregene Accessories (Qiagen), and PCR analyses were performed as follows. The primer pairs for detection of the junction region targeting the MI-MAC(1) were PGK5/G418 3AS (0.6 kb). The primer pairs for detection of the junction region targeting the MI-MAC(2) and the MI-MAC(3)+CrB were Bxb1 attR 2L/MICRISP Neo 2R (0.2 kb). The primer pairs for detection of the junction region targeting the MI-MAC(3) and the MI-MAC(2)+CrN were PGK Crisp L1/Bsd Crisp R1 (1.0 kb). The primer pairs for detection of the junction region targeting the MI-MAC(4) and the MI-MAC(4)+CrH were R4 attR 2L/MICRISPR Bsd 2R (0.2 kb). The primer pairs for detection of the junction region targeting the MI-MAC(5) and the MI-MAC(5) Sim were 3′HPRT L1/MI Hyg R1 (2.9 kb).

Sequencing to investigate the mutation of the DRGs was performed as follows. The primer pairs for detection of the Neo resistance gene, BS resistance gene, and Hyg resistance gene were PGK crisp L1/Neo Crisp R1 (0.6 kb), PGK crisp L1/Bsd Crisp R1 (1.0 kb), and Hyg Crisp L1/Hyg Crisp R1 (0.5 kb), respectively. The primer sequences are shown in Table 3.

**Table 3:** Primer sequence.

Primer	Sequence (5′-3′)
PGK Crisp L1	TCTCGTGCAGATGGACAGCA
Neo Crisp R1	CGGCAGGAGCAAGGTGAGAT
Bsd Crisp R1	GGGCAGCAATTCACGAATCC
Bxb1 attR 2L	GGCCGTGATGACCTGTGTCTT
MICRISP Neo 2R	TGTGCCCAGTCATAGCCGAAT
R4 att R 2L	TGTTCCCCAAAGCGATACCAC
MICRISPR Bsd 2R	GCTGGCGACGCTGTAGTCTTC
3’HPRT L1	GTGAATCTTTGTCAGCAGTTCCCTTTTA
MI Hyg R1	GCAAAGTGCCGATAAACATAACGATCT
PGK Crisp L1	TCTCGTGCAGATGGACAGCA
Neo Crisp R1	CGGCAGGAGCAAGGTGAGAT
Bsd Crisp R1	GGGCAGCAATTCACGAATCC
Hyg Crisp L1	GCAGCTTTGCTCCTTCGCTTT
Hyg Crisp R1	CAGGCAGGTCTTGCAACGTG

Cel-1 assays of CHO MAC1, MAC2 and 21HAC2 were evaluated using the Surveyor Mutation Detection Kits (IDT), in accordance with the manufacturer’s instructions. The primer pairs for detection of the NeoR mutation on CHO MAC1 were PGK Crisp L1/Neo Crisp R1. The primer pairs for detection of the Hyg R mutation on CHO MAC2 were Hyg Crisp L1/Hyg Crisp R1. The primer pairs for detection of the Bsd mutation on CHO 21HAC2 were Bsd Crisp L2/Bsd Crisp R1. The DNA of the transfected cells were used as temple DNA. The mutation rate was evaluated by ImageJ and the method for calculating indel frequency was as previously described [24]. The mutation of DRGs were evaluated by the Cel-1 assay and sequencing. The primer pairs for detection of DRG mutations were the same as used for junction PCR assays. PCR was performed using template DNA mixed at equal amounts, with wild-type and mutant-type sequences of DRGs.

### Luc assay

Luminescence assays were measured using a plate reader (Tecan infinite F500). For evaluation of Eluc luminous intensity and ATP activity, cell lysates were prepared using Pikka gene (Toyo INK. Group). Eluc luminous intensity and ATP activity were evaluated by mixing the cell lysate and luciferin (Toyo INK. Group) or CellTiter-glo (Promega), respectively. Luc assays of Cluc were assayed using Cypridine Luciferase Assay Kit (NEB) and evaluated by mixing the cell culture supernatant and Cypridina lucierin (NEB). Eluc and Cluc activities were normalized by dividing luminous intensity by ATP activity.

## Results

### Validation of mutation induction rate

**Fig 2:**
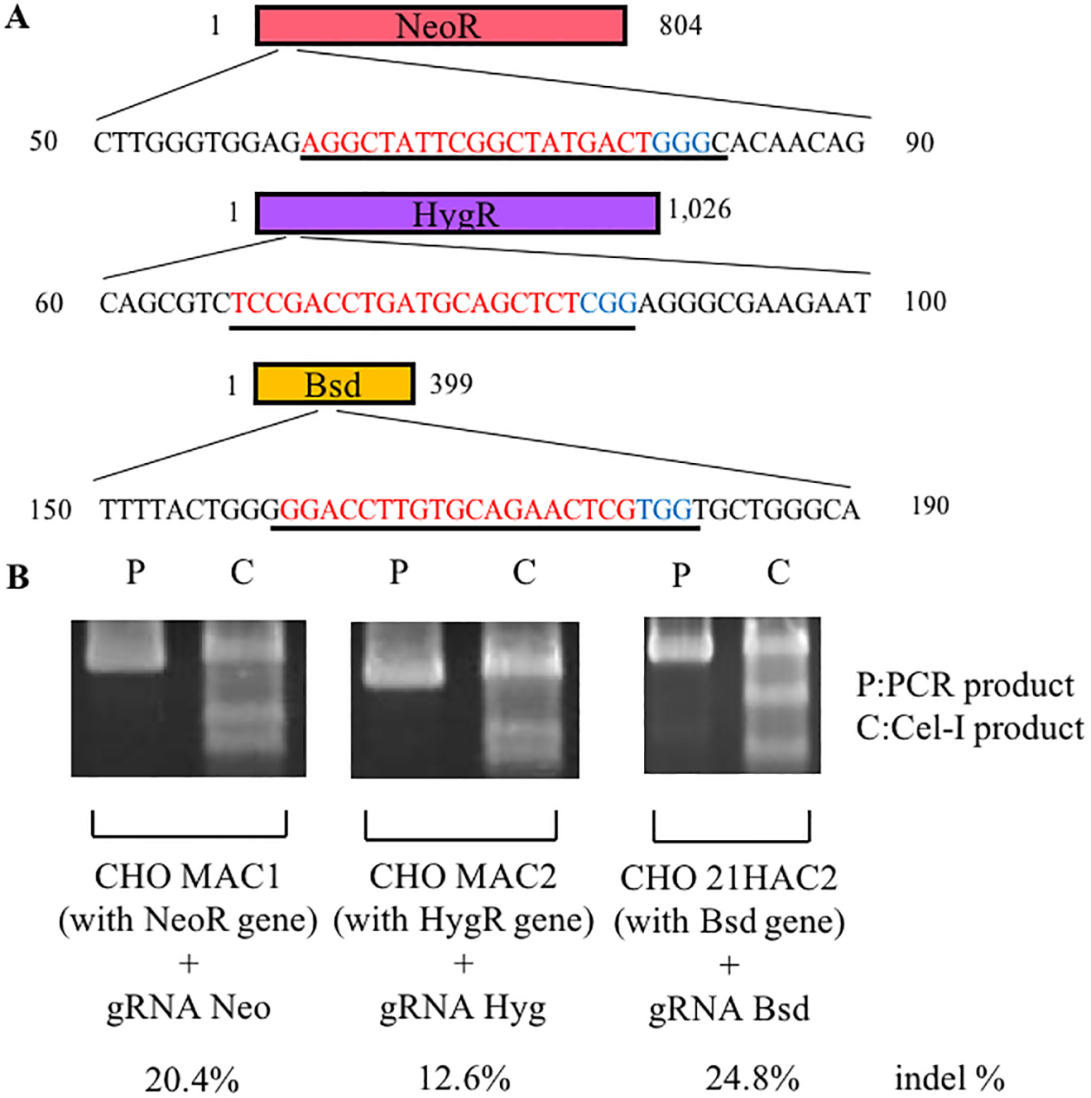
Efficiency of mutation induction by each gRNA. (A) Schematics of the drug resistance genes and targeted region of each gRNA. Underlines represent the gRNA recognition region, the PAM sequence (blue), and the target sequence (red). (B) Indel frequency in each drug resistance gene analyzed by Surveyor nuclease assay. Genomic DNA was extracted from CHO K1 cells carrying each drug resistance gene after transfection.

The MI system has multiple SSR systems (TP901, Bxb1, φC31, R4 attP, and FRT) for the efficient integration of multiple GLVs. However, as GLV loading onto the MI-MAC is performed, the number of available selective drugs decreases. To integrate multiple GLVs onto the MI-MAC using all SSR sites, we attempted to use the CRISPR-Cas9 system to knock out and reuse the DRGs. In this study, we chose three commonly used drugs (geneticin, blasticidin, and hygromycin) to select those cells in which the desired SSR had occurred. Fig 2A illustrates the sequence of three DRGs and the regions targeted by each gRNA (designated as gRNA Neo for the neomycin resistance gene, gRNA Bsd for the blasticidin resistance gene, and gRNA Hyg for the hygromycin resistance gene). Each gRNA was co-expressed with the Cas9 protein from the same plasmid vector (px330). To verify the efficiency of mutagenesis by each gRNA, the CHO cells carrying a HAC/MAC possessing each DRG (the CHO MAC1 cells carrying the neomycin resistance gene, the CHO MAC2 cells carrying the hygromycin resistance gene, and the CHO 21HAC2 cells carrying the blasticidin resistance gene) were transfected with the corresponding gRNA/Cas9 co-expression vector [2, 9]. At 3 days after transfection, genomic DNA was extracted and evaluated by the Cel-1 assay. The mutation induction rates of gRNA Neo, gRNA Hyg, and gRNA Bsd were 20.4%, 12.6%, and 24.8%, respectively (Fig 2B). Considering this result, we used these Cas9/gRNA co-expression vectors to knock out three DRGs in this study.

### Multiple GLV integration and knockout of DRGs

**Fig 3:**
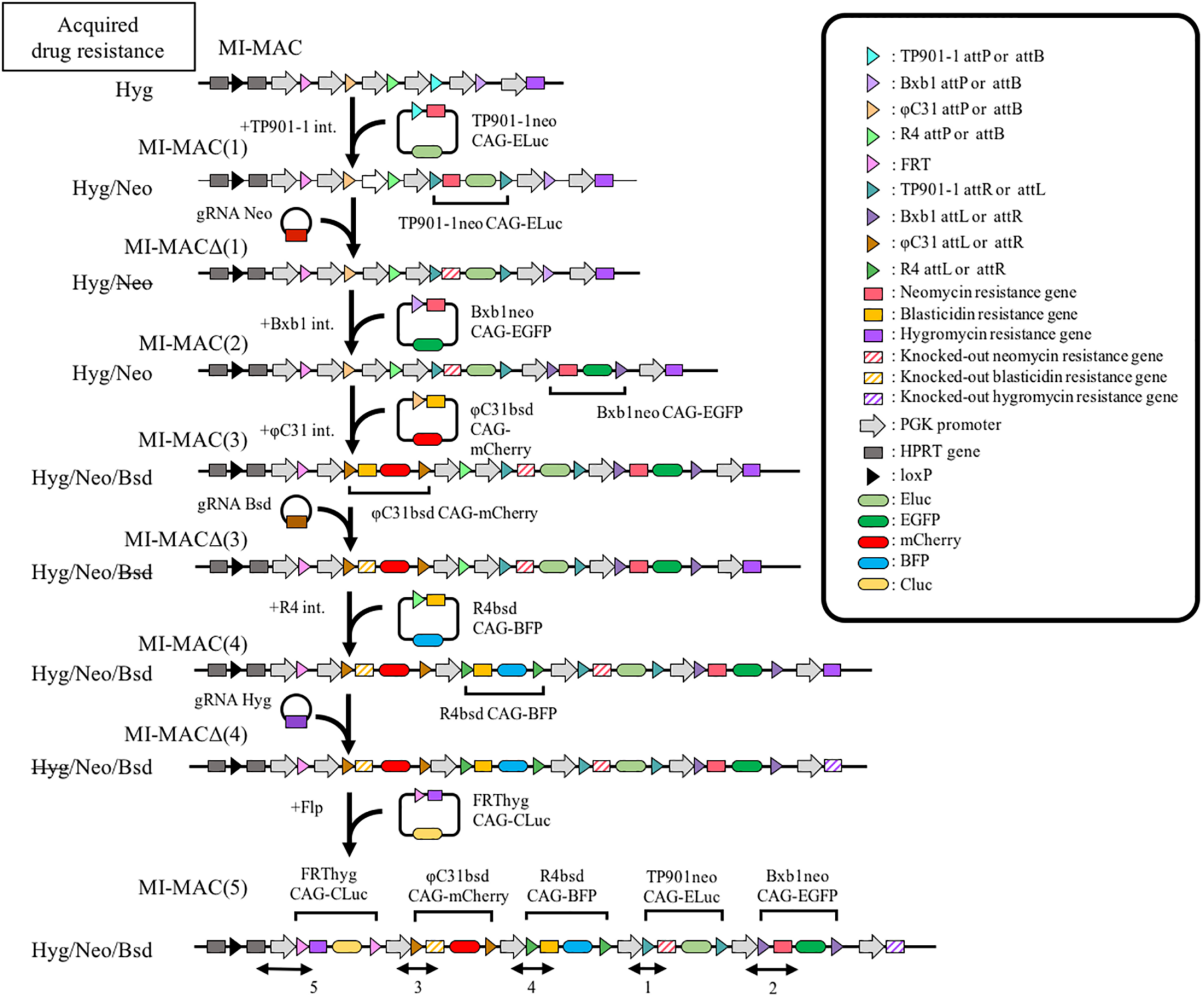
Multiple-GLV loading in CHO K1 cells carrying the MI-MAC by disruption of DRGs via the CRISPR-Cas9 system. Schematics of multiple-GLV loading. Transfections are carried out sequentially in accordance with the upper to lower illustrations. A transgene carrying a site-specific recombination (SSR) sequence (triangle) adjacent to the drug resistance gene (DRG, square), and subsequently a GOI (ellipse). SSRs are carried out between the MI-MAC and the GLV with the same integration system (depicted by same colors of triangle). Striped boxes depict the DRGs knocked out by the CRISPR-Cas9. Junction PCR is carried out covering the MI-MAC region and the transgene (bidirectional arrow). Acquired drug resistance genes on each MI-MAC are shown at the left side of the illustrate. The strikethrough represents the knock-out DRG.

We developed a new method to load multiple GLVs on the MI-MAC. In this method, GLV loading by the MI system and knocking out DRGs by the CRISPR/Cas9 system were carried out separately and sequentially in the following procedure (Fig 3, Table 1). The details of each resultant MI-MAC and transgenes are shown in Table 1. When the cells carrying each MI-MAC are sensitive to the selection drug for the next GLV integration, the next GLV loading is carried out directly. For example, because the CHO K1 cells carrying the MI-MAC are sensitive to geneticin, the first GLV (TP901-1neo CAG-Eluc) is loaded directly and the cells carrying the resultant MI-MAC (MI-MAC(1)) are selected using geneticin. On the other hand, when the CHO K1 cells carrying the MI-MAC are resistant to the selection drug for the next GLV integration, the DRG needs to be knocked out by the CRISPR-Cas9 system prior to the next GLV integration. After knocking out the DRG, the next GLV integration is selected by the drug corresponding to the knocked-out DRG. That is, because the CHO K1 cells carrying the MI-MAC(1) are resistant to geneticin, there is a need to knock out the neomycin resistance gene by the CRISPR-Cas9 (gRNA Neo) prior to the second integration to load the EGFP gene with geneticin selection. After knocking out the neomycin resistance gene (MI-MACΔ(1)), the second GLV (Bxb1neo CAG-EGFP) is integrated and the cells carrying the resultant MI-MAC (MI-MAC(2)) are selected using geneticin.

**Fig 4:**
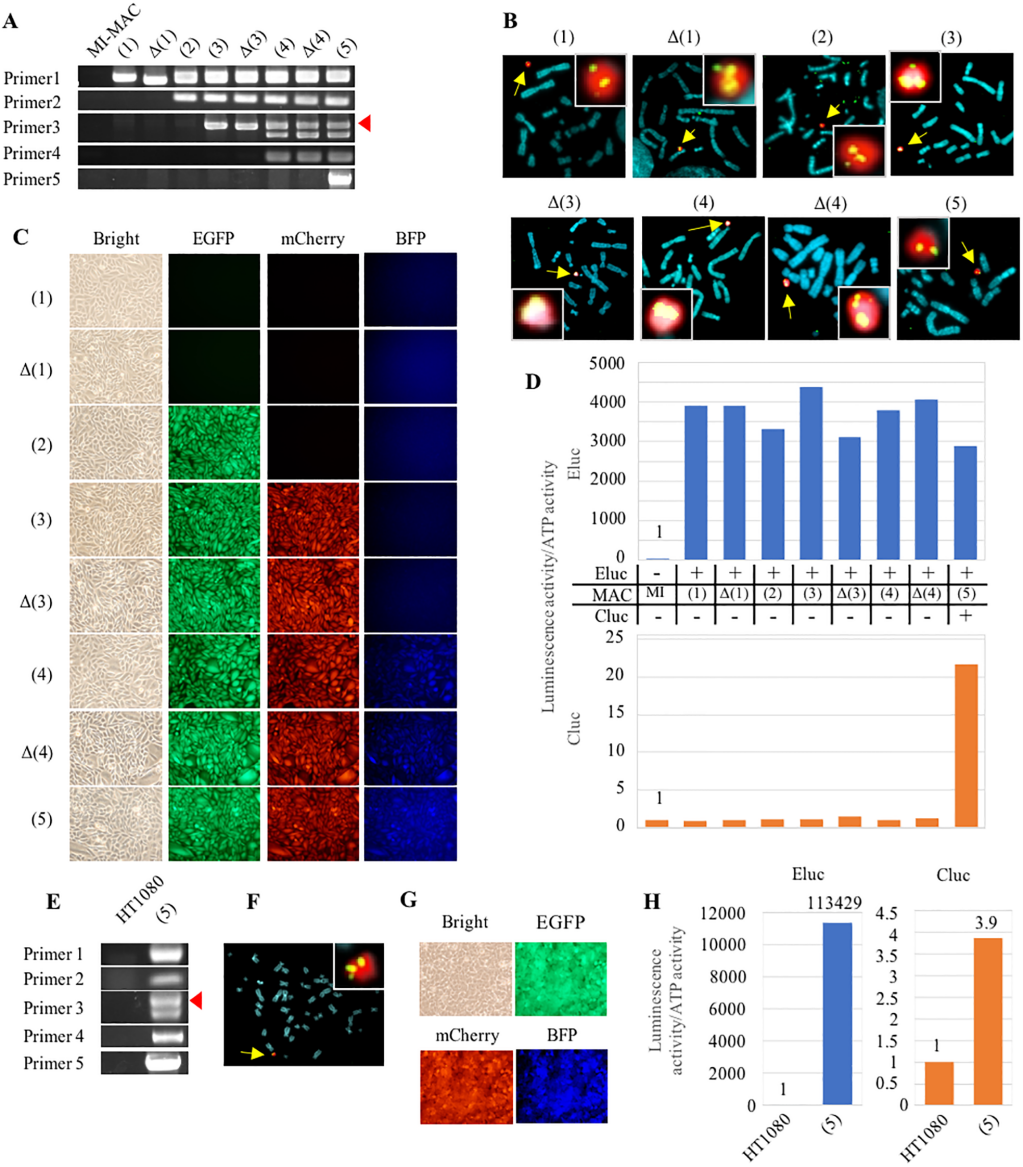
Multiple-GLV loading in CHO K1 cells carrying the MI-MAC and transfer of the MI-MAC with multiple GLV to HT1080 cells. (A) Junction PCR analyses of CHO K1 cells carrying each MI-MAC. The number of the primer pair corresponds to the bidirectional arrow of Fig 3. An arrowhead shows the expected band of primer 3. (B) Two-color FISH analyses of CHO K1 cells carrying each MI-MAC using the digoxigenin-labeled mouse Cot-1 probe (red) and biotin-labeled transgene probe (green). When CHO K1 cells are transfected with gRNA/Cas9 co-expression vector, a transgene transfected just prior to this is used as a biotin-labeled probe. Arrows show MI-MACs. The inset shows enlarged images of each MI-MAC. (C) Fluorescence image analyses in CHO K1 cells carrying each MI-MAC. Bright EGFP, mCherry and BFP micrographs are shown. (D) Luminescence assay in CHO K1 cells carrying each MI-MAC. Luminescence activities were normalized by dividing luminous intensity by ATP activity. The blue and orange bars represent Eluc and Cluc luminescence, respectively. On the panel between the two bar charts, the + and − represent the presence and absence of transgene on the MI-MAC, respectively. (E) Junction PCR analyses of HT1080 cells carrying each MI-MAC. Same primer pairs are used as in Fig. 4A. The arrowhead shows the expected band of primer 3. (F) Two-color FISH analysis of HT1080 cells carrying the MI-MAC(5) using the digoxigenin-labeled mouse Cot-1 probe (red) and biotin-labeled FRThyg CAG-Cluc probe (green). An arrow shows the MI-MAC(5). The inset shows enlarged image of the MI-MAC(5). (G) Fluorescence image analyses in HT1080 cells carrying the MI-MAC(5). Bright, EGFP, mCherry, and BFP micrographs are shown. (H) Luminescence assay in HT1080 cells carrying the MI-MAC(5). Eluc and Cluc activities were normalized by dividing luminous intensity by ATP activity. The blue and orange bars represent Eluc and Cluc luminescence, respectively.

As a proof of concept, in this study, we attempted to establish cells exhibiting three fluorescence signals and two luminescence ones (EGFP, mCherry, BFP, Cluc, and Eluc) by integrating the five GLVs at five SSR sites on the MI-MAC. To establish such cells, loading with five GLVs and the knockout of three DRGs were necessary. Therefore, we actually performed five gene integrations and three DRG disruptions step by step. To detect each expected recombination, the junction PCR primers were designed to cover a joint region within the MI-MAC region and the DRG of the GLV (Fig 3). Junction PCR analyses showed that each GLV was recombined at each SSR site as expected (Fig 4A). In addition, even when the loading of multiple GLVs and the knockout of multiple DRGs were repeated, there were no deficiencies in the recombination sequences that had already been integrated (Fig 4A). Among the clones resistant to each selection drug, the frequencies of integration into each SSR site, TP901-1, Bxb1, φC31, R4, and FRT, were 41.7% (10/24), 91.7% (22/24), 100% (24/24), 4.2% (2/48), and 29.2% (7/24), respectively (Table 1). Upon GLV integration, we knocked out the DRGs. Among the clones picked up, the frequencies of clones sensitive to each selection drug, geneticin, blasticidin, and hygromycin, were 3.1% (6/192), 16.7% (4/24), and 7.3% (7/96), respectively (Table 1). Sequencing of each DRG in representative clones that were drug-sensitive showed the following mutations: a 53-bp deletion in the neomycin resistance gene on the MI-MACΔ(1), a 16-bp deletion in the blasticidin resistance gene on the MI-MACΔ(3), and a 61-bp insertion in the hygromycin resistance gene on the MI–MACΔ(4). Next, FISH analyses confirmed that each MI-MAC was maintained independently from the host chromosome and that the transgene was integrated into the MI-MAC without randomly integration into the host chromosome (Fig 4B). These results showed that GLVs were loaded onto the MI-MAC as expected by employing the MI system and reusing the DRGs.

Fluorescence image analyses showed that the MI-MAC(2), the MI–MAC(3), and the MI-MAC(4) acquired EGFP, EGFP/ mCherry, and EGFP/ mCherry/BFP, respectively (Fig 4C). Luc assay showed that Eluc and Cluc on the MI-MAC(1)–(5) and the MI-MAC(5), respectively, were expressed functionally in each cell (Fig 4D). The luminescence intensity of Eluc in the CHO K1 cells carrying each MI-MAC was maintained stably. Fluorescence image analyses and Luc assay showed that the multiple GOIs on each MI-MAC were not silenced in the course of multiple-GLV loading and knocking out the DRGs. In summary, the CHO K1 cells carrying the MI-MAC(5) functionally expressed all three fluorescent proteins and the two luminescent ones. These results indicate that the method can load multiple GLVs without silencing or deletions, despite the multiple rounds of gene insertion by SSR and disruption by the CRISPR-Cas9.

### Transfer of the MI-MAC(5) with five GOIs into HT1080 cells

The HAC/MAC vectors are transferable to a variety of cells via MMCT. Therefore, to confirm that five GLVs are truly loaded on the MI-MAC(5) without integration into endogenous CHO K1 chromosomes, the MI-MAC(5) was transferred from CHO K1 cells to HT1080 cells via MMCT. Among the neomycin-resistant HT1080 cells into which the MI-MAC(5) was transferred, junction PCR was performed under the same conditions as for the CHO K1 cells with the MI-MAC, and all results of PCR analyses were shown to be positive (Fig 4E). FISH analysis showed that the MI-MAC(5) was independently maintained in the HT1080 cells (Fig 4F).

Fluorescence image analyses showed that EGFP, mCherry, and BFP were expressed in the HT1080 cells carrying the MI-MAC(5), as in the CHO K1 cells with the MI-MAC(5) (Fig 4G). Furthermore, Luc assay showed that Eluc and Cluc were expressed functionally (Fig 4H). Both sets of results demonstrated that the MI-MAC possessing multiple GLVs established by the method was transferrable to other cells by MMCT one time, and all GOIs on the MI-MAC(5) were functionally expressed in the recipient cells. These results strongly suggested that all SSRs to the MI-MAC are successfully carried out without random integration to the host chromosomes in CHO K1 cells. In conclusion, the method can load multiple GLVs using the SSR system and the CRISPR-Cas9 system separately, and the resultant MI-MAC is transferable and expresses the loaded genes in a functional manner.

### Multiple-GLV loading and knockout of DRGs by sequential integration with simultaneous disruption of DRG

We showed that the MI-MAC can possess and deliver five GLVs by using the SSR system and CRISPR-Cas9 system, and that the five genes are expressed functionally. However, the method has disadvantages in that it requires transfection, cloning and screening to be performed eight times for the loading of five GLVs. Therefore, the method needs more sessions of transfection than of gene loading. Moreover, drug selection for gene knockout is unavailable, so many cells including untransfected ones need to be analyzed.

**Fig 5:**
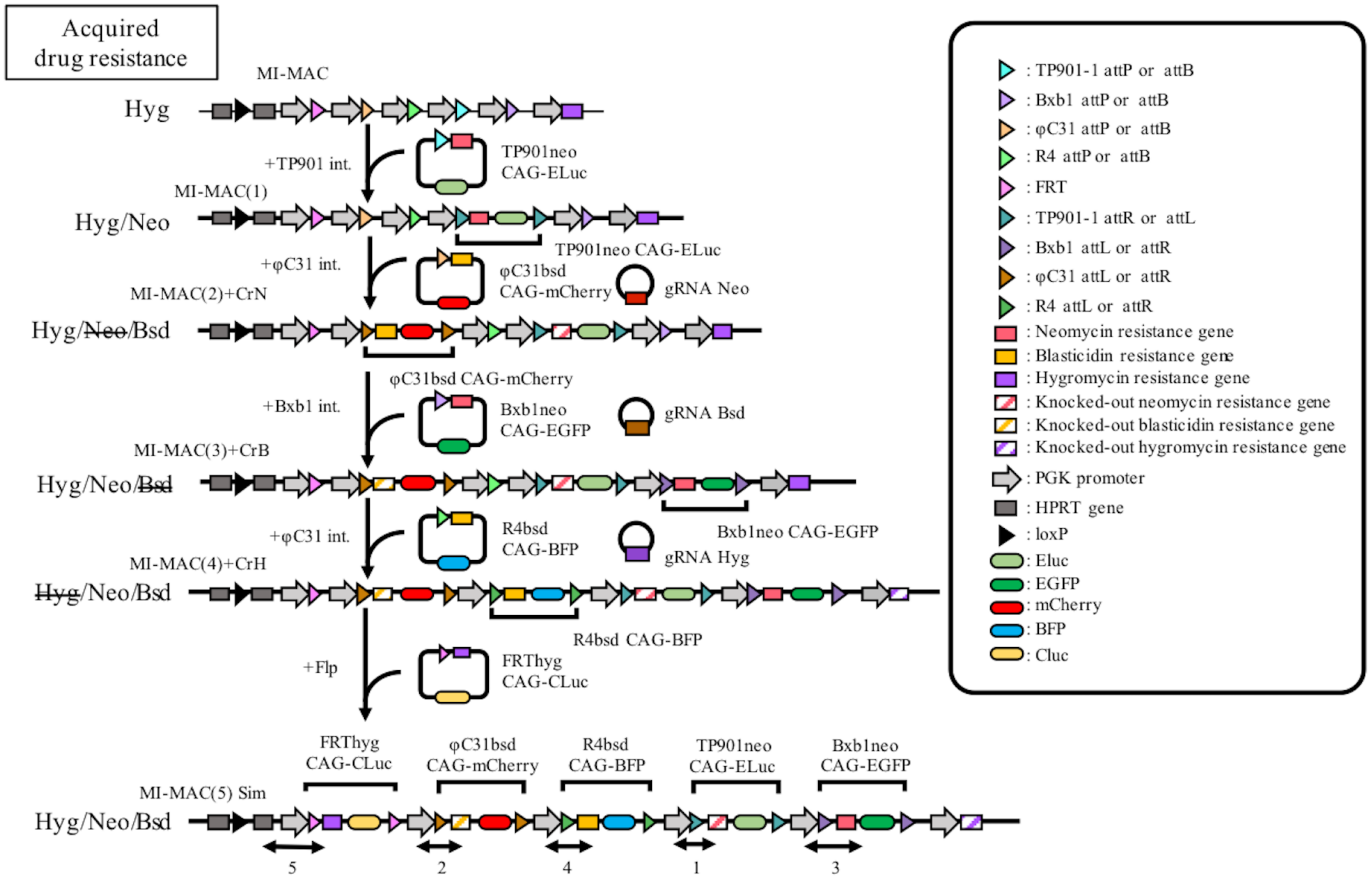
Multiple-GLV loading in CHO K1 cells carrying the MI-MAC using sequential integration with simultaneous disruption of DRG. (A) Schematics of multiple-GLV loading using sequential integration with simultaneous disruption of DRG. Transfections are carried out sequentially in accordance with the upper to lower illustrations. A transgene carrying a site-specific recombination (SSR) sequence (triangle) adjacent to the drug resistance gene (DRG, square), and subsequently a GOI (ellipse). SSRs are carried out between the MI-MAC and the GLV with the same integration system (depicted by same colors of triangle). Striped boxes depict the DRGs knocked out by the CRISPR-Cas9. Junction PCR is carried out covering the MI-MAC region and the transgene (bidirectional arrow). Acquired drug resistance genes on each MI-MAC are shown at the left side of the illustration. The strikethrough represents the knock-out DRG.

To solve these problems, we attempted to improve the former original method by loading GLVs and knocking out the DRGs at the same time, for which a system involving sequential integration with simultaneous disruption of DRG was designed. Since this improved method employs a selection drug for screening the GLV-inserted cells, untransfected cells are mostly eliminated. In the improved method, GLV loading onto each MI-MAC is carried out using a selection drug to which CHO K1 cells carrying each MI-MAC are sensitive; at the same time, functional DRG on the MI-MAC is knocked out by the CRISPR-Cas9 system (Fig 5). For example, the CHO K1 cells carrying the MI-MAC(1) are resistant to geneticin and hygromycin, GLV loading for mCherry (φC31bsd CAG-mCherry) is selected using blasticidin, and at the same time knockout of the neomycin resistance gene is carried out by gRNA Neo. When knockout is not necessary for GLV loading, GLV is loaded in the same way as in the original method, for example, the establishment of the MI-MAC(5) Sim (Fig 5, Table 2).

**Fig 6:**
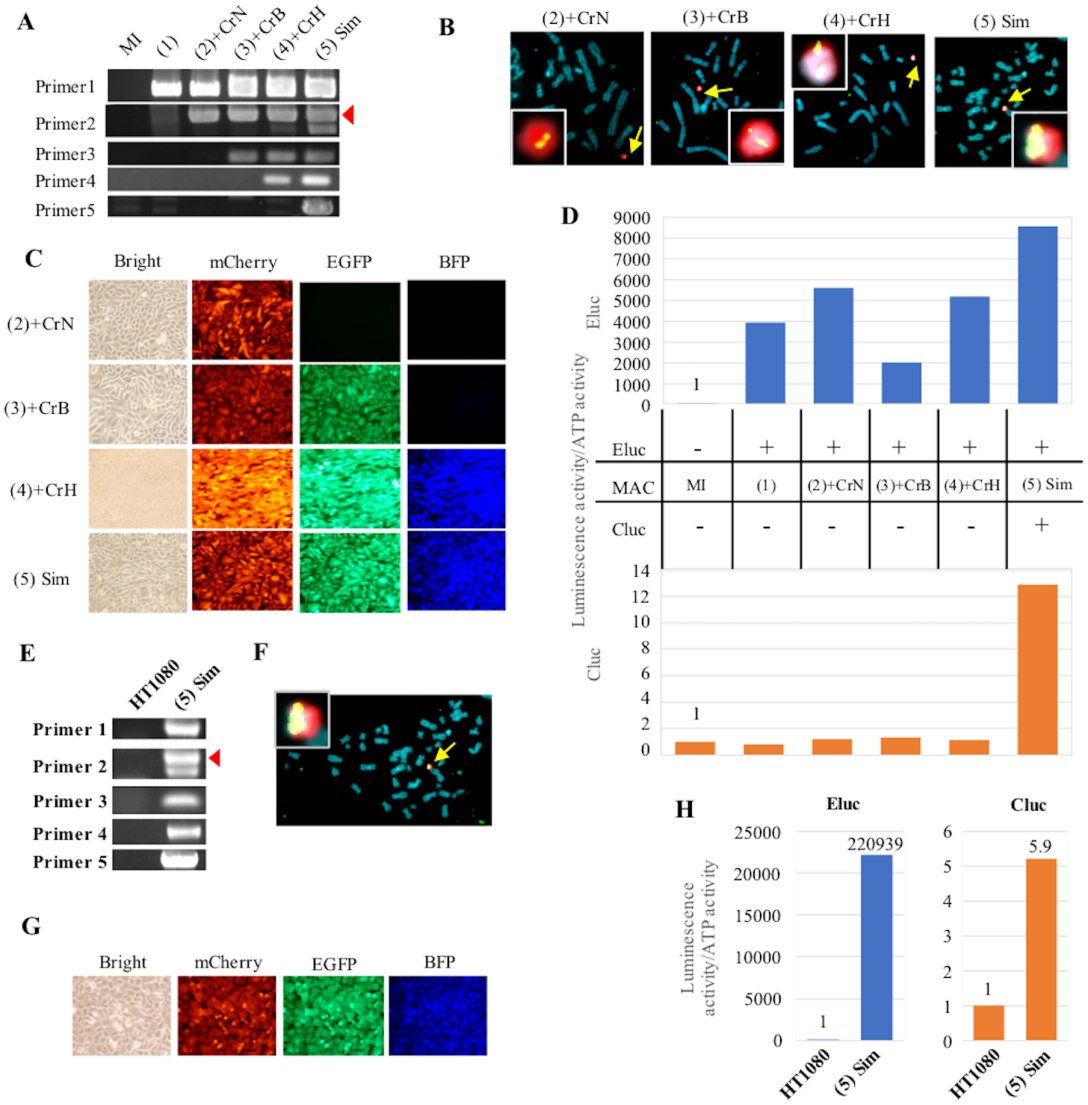
Multiple-GLV loading in CHO K1 cells by sequential integration with simultaneous disruption of DRG and transfer of the MI-MAC with multiple GLVs to HT1080 cells. (A) Junction PCR analyses of CHO K1 cells carrying each MI-MAC. The number of the primer pair corresponds to the bidirectional arrow of Fig 5. An arrowhead shows the expected band of primer 2. (B) Two-color FISH analyses of CHO K1 cells carrying each MI-MAC using the digoxigenin-labeled mouse Cot-1 probe (red) and biotin-labeled transgene probe (green). Arrows show MI-MACs. The insets show enlarged images of each MI-MAC. (C) Fluorescence image analyses in CHO K1 cells carrying each MI-MAC. Bright, mCherry, EGFP and BFP micrographs are shown. (D) Luminescence assay in CHO K1 cells carrying each MI-MAC. Luminescence activities were normalized by dividing luminous intensity by ATP activity. The blue and orange bars represent Eluc and Cluc luminescence, respectively. On the panel between the two bar charts, + and − represent the presence and absence of transgene on the MI-MAC, respectively. (E) Junction PCR analyses of HT1080 cells carrying the MI-MAC(5) Sim. Same primer pairs are used as shown in Fig. 6A. An arrowhead shows the expected band of primer 2. (F) Two-color FISH analysis of HT1080 cells carrying the MI-MAC(5) Sim using the digoxigenin-labeled mouse Cot-1 probe (red) and biotin-labeled-FRThyg CAG-Cluc probe (green). An arrow shows the MI-MAC(5) Sim. The inset shows enlarged images of the MI-MAC(5) Sim. (G) Fluorescence image analyses in HT1080 cells carrying the MI-MAC(5) Sim. Bright, mCherry, EGFP, and BFP micrographs. (H) Luminescence assay in HT1080 cells carrying the MI-MAC(5) Sim. Eluc and Cluc activities were normalized by dividing luminescence by ATP activity. The blue and orange bars represent Eluc and Cluc luminescence, respectively.

To establish cells carrying the expected MI-MAC with GLVs loaded at the SSR site and knockout of the DRG, the following procedures were carried out. First, co-transfection with three vectors (GLV, integrase expression vector, and gRNA/Cas9 co-expression vector) was performed. Then, drug-resistant clones were selected using the selection drug. Next, the knockout of the DRG was detected using the selection drug corresponding to the gRNA after cloning. Cells established by these procedures will theoretically carry the MI-MAC in which the GLV is loaded and the DRG is knocked out. Among the drug-resistant clones, the rates of knocked-out clones were 7.8% (15/192) for the neomycin resistance gene on the MI-MAC(2)+CrN, 21.4% (6/28) for the blasticidin resistance gene on the MI-MAC(3)+CrB, and 88.2% (15/17) for the hygromycin resistance gene on the MI-MAC(4)+CrH. This result shows the improvement tendency of the frequency of knocking out of the DRGs, neomycin resistance gene (3.1% to 7.8%), blasticidin resistance gene (16.7% to 21.4%), and hygromycin resistance gene (7.3% to 88.2%) (Tables 1 and 2). Subsequently, these cells were further analyzed. Junction PCR analyses were carried out under the same conditions as used in Fig 3A, and it was shown that GLVs had integrated onto each SSR site of the MI-MAC, as expected (Fig 6A). In addition, it was confirmed that the recombined sequence was maintained without a deletion in the vicinity (Fig 6A). The rates of efficiency of GLV integration into each SSR site (φC31, Bxb1, R4, and FRT) were 100% (15/15), 100% (6/6), 26.7% (4/15), and 12.5% (3/24), respectively (Table 2). Moreover, sequencing of the representative clones showed a 2-bp deletion in the neomycin resistance gene on the MI-MAC(2)+CrN, a 2-bp deletion in the blasticidin resistance gene on the MI-MAC(3)+CrB, and a 1-bp insertion in the hygromycin resistance gene on the MI-MAC(4)+CrH. These results suggested that knockout of the DRGs can be carried out with GLV integration by SSR.

FISH analyses of each MI-MAC confirmed that each MI-MAC was independently maintained in the host cells without random integration (Fig 6B). The results also suggested that the improved method can indeed be used to integrate GLV and knock out the DRG at the same time.

Fluorescence image analyses showed that the MI-MAC(2)+CrN, the MI–MAC(3)+CrB, and the MI-MAC(4)+CrH acquired mCherry, mCherry/EGFP, and mCherry/EGFP/BFP, respectively (Fig 6C). Moreover, the CHO K1 cells carrying the MI-MAC(5) Sim exhibited three fluorescence signals of mCherry, EGFP, and BFP. Luc assay showed that Cluc is functionally expressed in the MI-MAC(5) Sim. In addition, the CHO K1 cells carrying each MI-MAC expressed Eluc stably (Fig 6D). From the results of both fluorescence imaging and Luc assay, five genes on the MI-MAC were shown not to be silenced.

We demonstrated that the improved method can load multiple GLVs on the MI-MAC via fewer transfections than in the original method. Furthermore, to the best of our knowledge, this is the first report of a method that was demonstrated to enable GLV loading by SSR and knockout by the CRISPR/Cas9 system at the same time on a mammalian artificial chromosome vector.

### Transfer of the MI-MAC(5) Sim to HT1080 cells

To verify that all GLVs were loaded on the MI-MAC (MI-MAC(5) Sim), the MI-MAC(5) Sim was transferred from CHO K1 cells to HT1080 cells via MMCT. The geneticin-resistant HT1080 cells were cloned and analyzed. Junction PCR under the same conditions as for the CHO K1 cells showed that all were positive (Fig 6E). FISH analysis showed that the MI-MAC(5) Sim was maintained independently in HT1080 cells without integration or translocation to the host chromosomes (Fig 6F).

Fluorescence image analyses showed that mCherry, EGFP, and BFP were expressed concurrently in the HT1080 cells (Fig 6G). In addition, Luc assay showed that both Eluc and Cluc were functionally expressed (Fig 6H). These results demonstrated that all GOIs on the MI-MAC(5) Sim were inserted into the expected SSR sites of the MI-MAC and were functionally expressed stably in other cells. These results proved that all GLVs on the MI-MAC(5) Sim were loaded as expected without random integration. In conclusion, the improved method can load multiple GLVs by employing the MI system and the CRISPR-Cas9 system at the same time and the resultant MI-MAC(5) Sim is transferable and expresses the transgenes in a functional manner.

## Discussion

The development of multiple-GLV-loading methods is expected to be beneficial in various fields of study, such as multiple-gene humanized models, multiple-gene monitoring models, disease models, reprogramming, and inducible gene expression systems, so various reports about such systems have been published. Kameyama et al. reported a multiple-GLV integration system that can load multiple GLVs to the modified region in advance by applying the Cre-loxP system [25]. In this system, part of the loxP sequence is modified, producing mutant loxP, and the combination of some mutant loxP sequences enables repeated integration. Since this system is based on the host chromosomes, it is only available for cells that have been modified in advance. If this system is applied to HACs/MACs, its ability to be used as a multiple-GLV-loading system can be expanded to various fields of study.

In this study, we developed new GLV-loading methods that are applicable to the MI-MAC. We loaded five GLVs (EGFP, mCherry, BFP, Eluc, and Cluc) and knocked out three DRGs on the MI-MAC by both methods. These GOIs were not only stably expressed throughout the multiple-GOI loading, but also almost homogeneously expressed in the clonal population. Furthermore, the MI-MAC possessing five GOIs is transferrable to other cells and all five representative genes are functionally expressed similarly in CHO K1 cells. In this study, we loaded multiple plasmid vectors (about 10 Kb each), although we have demonstrated that large genomic DNA can be loaded to the MI-MAC, for example, PACs (29 and 39 Kb) onto the MI-MAC [13]. These facts suggest that multiple GLVs containing large genomic DNA can be loaded on the MI-MAC by employing the multiple-GLV-loading methods.

Because integrases mediate irreversible integration, loading of additional SSR sites to the MI-MAC is theoretically capable of indefinite gene loading. However, repeated use of DRGs may hamper the identification of gene loading and disruption of DRG via PCR-based assays.

Generally, the amounts of time and labor required for loading multiple GLVs are greatly influenced by the convenience of the cell culture and the efficiency with which genes can be transferred into host cells. For example, use of a multiple-gene monitoring system in human iPS cells or ES cells may make it possible to trace the differentiation in multiple stages. However, if we attempt to load multiple genes into human iPS cells or ES cells by conventional methods, the following problems can be encountered. (1) High levels of labor and cost required for cell culture: Since human iPS and ES cells require maintenance to retain an undifferentiated state, feeder cells and reagents such as bFGF are required, which increase the labor and cost [26, 27]. (2) Low transfection efficiency: Since the efficiency of transfer of genes into human iPS cells and ES cells is lower than that into CHO K1 cells, it is more labor-intensive and expensive [28]. (3) Risk of karyotypic abnormality: The longer the culture of human iPS cells and ES cells is performed, the greater the risk of karyotypic abnormality [29]. Therefore, when loading multiple genes continuously, it is necessary to perform karyotype analysis in each step of genetic modification. (4) Risk of off-target issues with the CRISPR-Cas9 system: Since genome editing might cause unexpected mutations, more effort is required to eliminate the possibility of off-target effects on host genomes. (5) Positional effect from the host chromosome: In the conventional method, transgenes are affected by the chromosomal position on the host chromosome because of host chromosome-based gene loading. Therefore, even if differentiation induction is performed, the genes to be monitored may not be expressed due to silencing. These problems can be solved by the MI-MAC/HAC- and CRISPR-Cas9-mediated gene integration in CHO K1 cells. The CHO K1 cells are (i) immortalized and can be cultured in a medium with a conventional composition. In addition, (ii) the transfection efficiency of CHO K1 cells is higher than that of human iPS cells and ES cells. Moreover, since GLV loading is performed by SSR systems of the MI system, gene targeting can be carried out with higher efficiency than conventional targeting by homologous recombination. (iii) CHO K1 cells are available as donor cells for MMCT. Then, MI-MACs possessing multiple GLVs constructed in CHO K1 cells are transferable to desired cells. Therefore, the resultant cells are transferred multiple genes at a single time. Furthermore, (iv) since genome editing is carried out in CHO K1 cells that just act as mediators, there is no problem if unexpected mutations arise on the host chromosomes of CHO K1 cells due to off-target effects. Finally, (v) because the MI-MACs are maintained independently from host chromosomes, multiple genes on the MI-MACs are not influenced by positional effects from host chromosomes, which eliminates the possibility of silencing. Considering the above, there are many advantages of using the MI-MAC to introduce multiple genes to desired cells.

In this study, we have reported that new methods can load multiple GLVs on the MI-MAC, but some technical improvements are still required. For example, the R4 integration system had lower efficiency of integration than the other SSR systems. However, Yamaguchi et al. and Yoshimura et al. reported higher efficiency for an R4 integration system. This difference in efficiency might be due to various factors, such as the difference of genes on the GLVs, clonal variation of the cells, the use of different combinations of integration system and DRG, and the expression level of R4 integrase. The examination of these differences is expected to improve the efficiency of the R4-mediated integration of MI-MACs. Concerning knockout, the improved method can increase the purity of the transfected cells to some extent by eliminating the untransfected cells through selection with the corresponding selection drug. Furthermore, it is expected that an improvement of transfection efficiency of gRNA and Cas9 co-expression vectors, using viral vectors, might reduce the labor and time required for multiple-GLV loading.

For five GLV loading, the original method needs eight steps of transfection, cloning, and analyses. We reduced the process to five steps in the improved method, but this method has potential for further improvement. Since the MI system can theoretically load two or more GLVs in one transfection, the steps of the method may be reduced for efficient multiple-gene loading. For example, the first step is co-integration of NeoR-ELuc and Bsd-mCherry with simultaneous disruption of the HygR gene. The second step is integration of HygR-CLuc with co-disruption of the NeoR gene and Bsd. The third step is co-integration of NeoR-EGFP and Bsd-BFP. In this way, the process may be reduced to three steps.

These multiple-GLV-loading methods are expected to solve the problems associated with conventional versions of such methods. Consequently, these methods should contribute to various fields of study, such as new drug discovery by developing multiple gene humanized models of drug metabolism-related genes, detailed analyses of induction of the differentiation of pluripotent stem cells by monitoring multiple GOIs, and high production of antibodies by using multiple copies of the genes that produce them.
